# Surgical repair of prolapsed stoma via the buttonpexy approach: a case series

**DOI:** 10.1093/jscr/rjaf1069

**Published:** 2026-01-16

**Authors:** Fatima Al Zahra, Chandni Nawaz, Muhammad Abdullah, Aleena Ihtasham, Alishah Haider, Hassan Amin

**Affiliations:** Department of Pediatric Surgery, King Saud Hospital, King Saud Road, Al-Salam District, Unaizah, Al-Qassim Region, Unaizah, 51911, Saudi Arabia; Department of Pediatric Surgery, Women’s and Children’s Hospital, 72 King William Road, North Adelaide, South Australia, Adelaide, 5006, Australia; Bahria University Medical & Dental College, Bahria Medical University Campus, Sailor St, Karachi, 74400, Pakistan; King Edward Medical University, Lahore, Punjab, 54000, Pakistan; University of Aberdeen, Aberdeen, Scotland, AB24 3FX, United Kingdom; Bahria University Medical & Dental College, Bahria Medical University Campus, Sailor St, Karachi, 74400, Pakistan

**Keywords:** stoma prolapse, buttonpexy, pediatric surgery, colostomy, ileostomy, case series

## Abstract

Stoma prolapse is a frequent complication following stoma formation in pediatric patients and often necessitates surgical intervention. The buttonpexy technique offers a minimally invasive, low-cost, and anesthesia-sparing alternative to formal stoma revision. This case series highlights its safety and effectiveness in managing pediatric stoma prolapse. Five pediatric patients with stoma prolapse secondary to various underlying conditions, including anorectal malformation, Hirschsprung’s disease, and Currarino syndrome, were managed using the buttonpexy technique. In each case, the prolapsed bowel was gently reduced, and pledgets derived from intravenous tubing were secured above and below the skin margins to anchor the stoma locally. The procedures were performed under local anesthesia, and all patients tolerated them well. Four patients demonstrated complete resolution without recurrence during follow-up, while one experienced partial prolapse that was successfully corrected with a repeat buttonpexy. None of the patients required conversion to formal stoma revision, and no complications such as infection, necrosis, or bleeding were observed. Follow-up ranged from one to eight months, confirming sustained stoma stability until definitive closure. The buttonpexy technique is a simple, safe, reproducible method for managing pediatric stoma prolapse. It can be performed under local anesthesia, minimizing anesthetic exposure and hospital stay. This approach provides a practical first-line option before considering formal revision, especially in resource-limited or pediatric settings where minimizing surgical and anesthetic risks is paramount.

## Introduction

A stoma is an undesirable consequence of a celiotomy made in dire circumstances to save the patient from undesirable, grave consequences. It is generally associated with a number of complications: stricture, prolapse, adhesions, excoriation, bleeding, and parastomal herniation [1, 2]. All of these pose a risk to the patient in terms of hospital admission and need for surgical intervention. To address the issues of prolapse, we have started to perform the buttonpexy technique for local anchorage. The idea is to use pledgets derived from intravenous tubing and sew them above and below the skin to provide a barrier that cuts off the wave and keeps the gut anchored to the margins of the stomal window. The rationale is to reduce the cost, hospital length of stay, anesthesia exposure, and buy time till closure of the stoma. Here we present five cases of prolapse managed by the buttonpexy technique at our setup and their results. This manuscript was prepared following the CARE guidelines.

## Case 1

**Figure 1:**
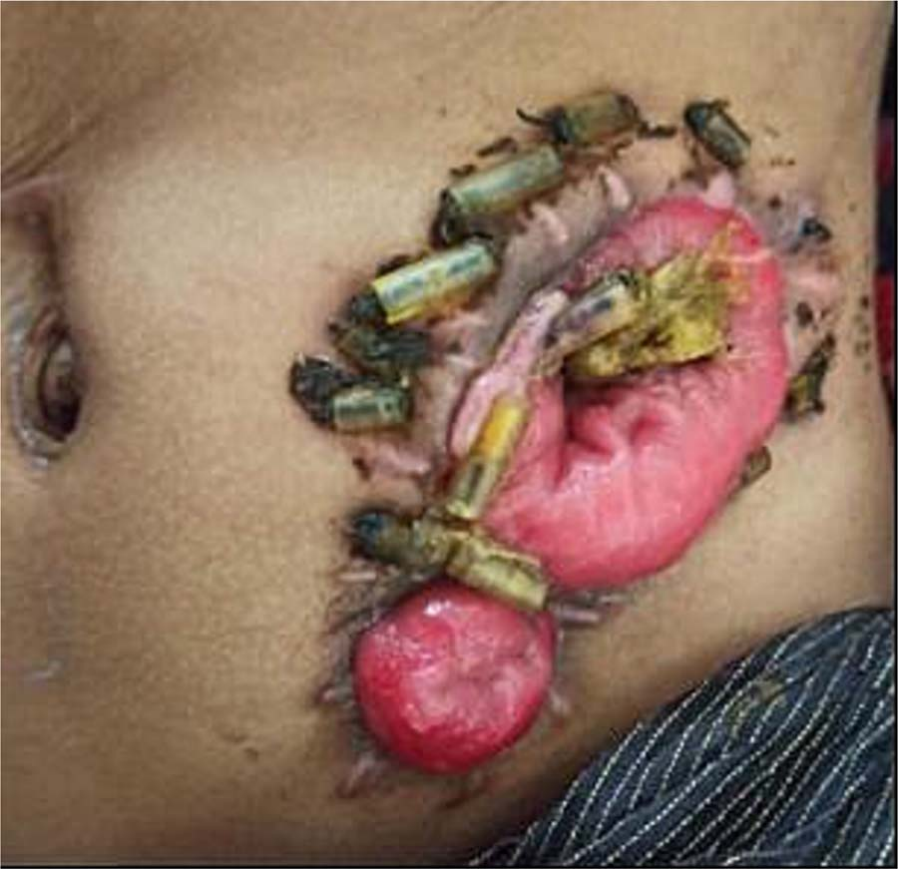
Status at one-month follow-up in a 3-year-old female with anorectal malformation after buttonpexy repair of a prolapsed divided descending colostomy. One-month follow-up after buttonpexy repair.

A 3-year-old female child was a case of anorectal malformation with rectoperineal fistula. The child had a divided descending colostomy in place that was made at the age of eight months. She presented with progressive prolapse of the proximal stoma, which measured over 10 cm at the time of assessment. The diagnosis of stoma prolapse was made clinically based on inspection and examination findings; no imaging or laboratory tests were required. Buttonpexy was performed under local anesthesia. The stoma was reduced gently and pledgets derived from IV tubing were sewn above and below the skin margins. The child tolerated the procedure well. Follow-up at one-month post-procedure showed no recurrence. A follow-up, a month later again showed that it was holding up nicely (Fig. 1).

## Case 2

A two-month-old infant with Currarino syndrome had previously undergone a transverse loop colostomy under local anesthesia in the neonatal period. The patient presented with visible stoma prolapse that interfered with stoma care. The prolapse was diagnosed clinically, and the buttonpexy technique was used for correction. Partial recurrence occurred a few days later, which was managed successfully with a redo buttonpexy. The stoma remained stable until reversal following the definitive procedure.

## Case 3

**Figure 2:**
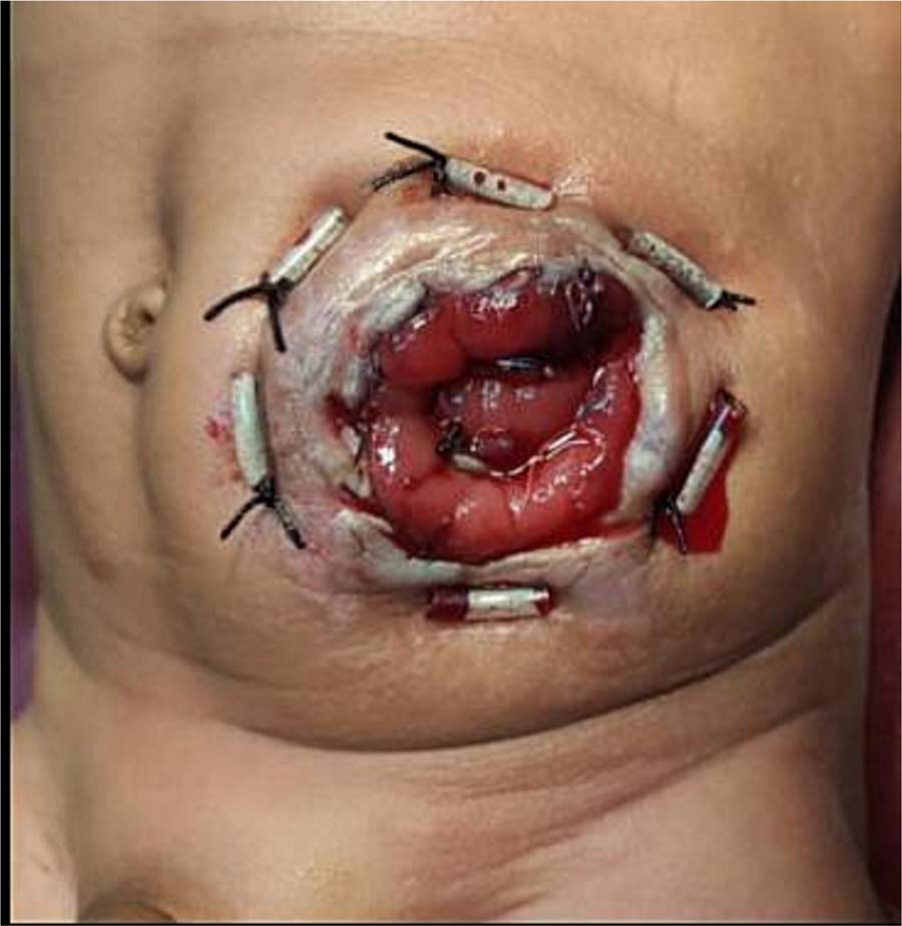
Postoperative appearance following buttonpexy repair in a 4.5-month-old child with Hirschsprung’s disease. Postoperative view showing buttonpexy fixation.

A 4.5-month-old child with Hirschsprung’s disease had undergone an emergency transverse loop colostomy at three months of age due to persistent abdominal distension unresponsive to conservative management. The patient presented with a prolapsed stoma diagnosed clinically on examination. Buttonpexy was performed under local anesthesia with successful reduction and fixation of the prolapsed segment. The child recovered well, and follow-up examinations revealed no recurrence (Fig. 2).

## Case 4

**Figure 3:**
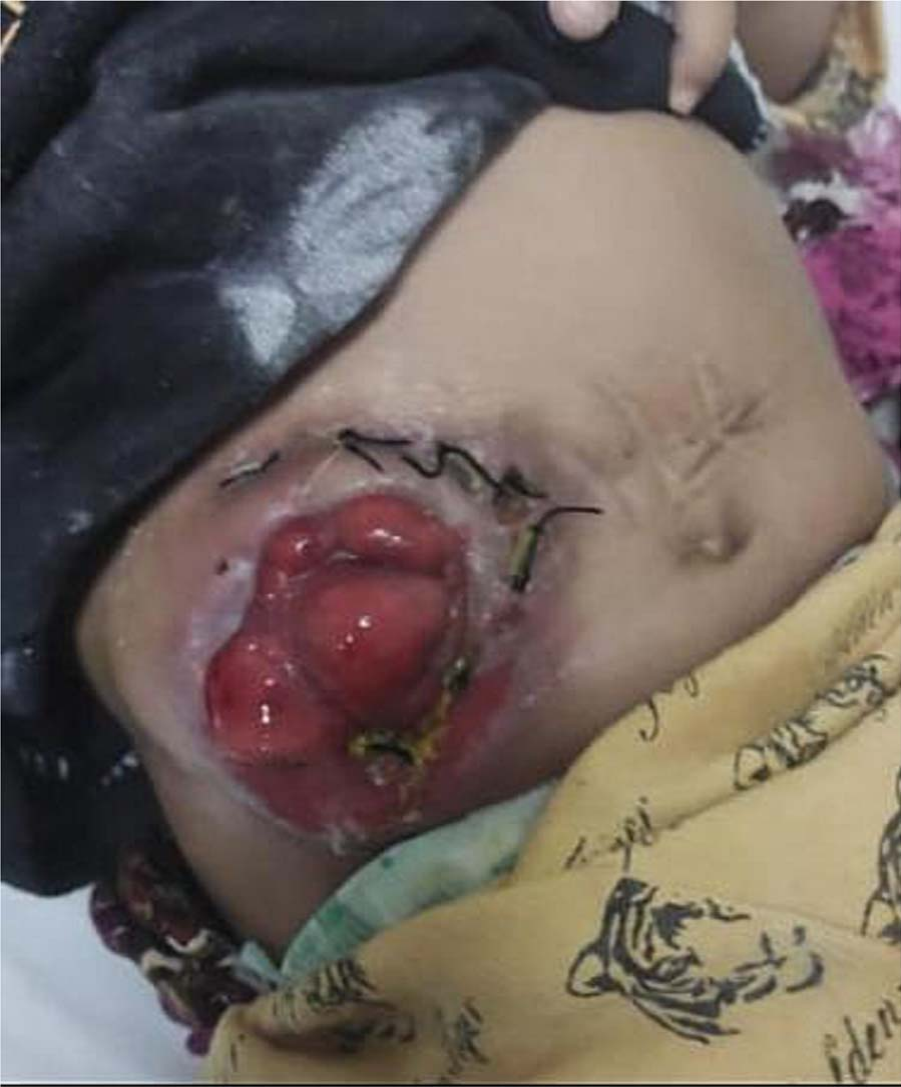
Follow-up at one month after buttonpexy repair in an 8-month-old child with Hirschsprung’s disease. The stoma remains stable with no recurrence of prolapse. One-month follow-up showing well-healed stoma.

An eight-month-old child with Hirschsprung’s disease had previously undergone a transverse divided colostomy at four months of age. The patient presented with prolapse of the distal stoma loop, which was confirmed clinically. Under local anesthesia, the prolapsed segment was reduced and secured using the buttonpexy method. The postoperative course was uneventful, and follow-up at one month demonstrated satisfactory stoma appearance with no evidence of recurrence (Fig. 3).

## Case 5

A five-month-old male presented with a chronically prolapsed loop stoma created at two months of age in Azad Kashmir for Hirschsprung’s disease. The prolapse was clinically evident on examination. Buttonpexy was performed under local anesthesia, and the stoma maintained good fixation until definitive closure was carried out approximately eight months later. No complications were noted during the follow-up period.

## Discussion

Many pediatric diseases require stoma formation under emergency or elective settings. If associated with subsequent complications, it needs to be addressed, requiring its own management procedure. Exposing the developing brain to anesthesia has been well proven to have deleterious effects on myelination and overall cognition of the child. The stomas made in the pediatric age group are usually temporary and are mostly reversed after gut rest, hemodynamic stability, and a definitive procedure. Adding a procedure to deal with stoma setbacks is not an ideal situation in this group of patients.

We encounter several patients in our outpatient department and emergency department with a complaint of stoma prolapse along with other stoma complications. Sometimes it is acute and incarcerated, and applying sugar to reduce edema and facilitate reduction is a common practice, but in chronic cases, a definitive approach is needed [3]. Prolapse is not only agitating for the patient and caregiver but also predisposes the hanging intestinal loop to trauma by exposure, redundancy, and entrapment in clothing or wheelchairs in neurologically impaired children. The loop stoma and divided stoma have an altered tendency to prolapse, as in the loop stoma, only the anterior wall is opened, and gut continuity is intact on the posterior wall; the peristalsis is not hindered and leads to prolapse of the loop through the anterior breached surface. Though a divided stoma is not susceptible to prolapse to the same extent, yet it still can prolapse especially the proximal diverting loop, with intact integrated peristalsis.

The location of the stoma formation also contributes to the length of the hanging segment predisposes to prolapse, as is the case in transverse stomas. The stoma made in a rectosigmoid region is often anchored between the splenic flexure and left peritoneal reflection above and below by an intraperitoneal rectal segment and therefore has lesser rate of prolapse compared to the transverse stoma. Another factor is the disease process contributing to the need of stoma formation. Conditions like Meconium ileum, Hirschsprung's disease, etc. are associated with aberrant propulsion due to inherent biochemical and neuronal changes and may also confer added incidence. Keeping these factors in mind, we aimed to find ways to intervene minimally between the scheduled staged procedures. It’s a norm to address the prolapse patients with the revision if it is leading to problems in its management. We started to perform buttonpexy for local anchorage and to counter the prolapse after reduction, as previously described by Canil *et al.* [4].

The buttonpexy is a safe, easy, cheap, and replicable procedure with no learning curve or downtime. It can be performed under local anesthesia and effectively combats the ongoing peristaltic waves. There are other methodologies introduced in the literature as well, like using a stapler to resect the redundant margins, mesh repair, doing stomal revision under local anesthesia, and using a purse string [5–8]. All of these have their own sets of pros and cons and have been utilized at intervals on the surgeon's discretion. However, considering our experience, we intend to introduce the buttonpexy technique in our setup in replacement of formal revision as a first-line treatment that may be followed by formal revision if the results are not optimal.

Among our cases, four patients were successfully managed with buttonpexy. The one case having partial prolapse was addressed by redo, and that sufficed. We experienced that it was safe and easy to perform and could be done under local anesthesia as a day case. The patients tolerated the procedure well. A minimal learning curve was required as compared to other techniques. In two patients, the buttonpexy did not manage to hold the prolapse; however, we believe several additional patient factors could have contributed towards recurrence. The results would become clearer with further experience.

The strength of this case series is demonstrating the practical applicability of the buttonpexy technique as a simple, low-cost technique which does not require general anesthesia for managing pediatric stoma prolapse. These cases demonstrate its reproducibility and favorable outcomes short term across different underlying diagnoses. However, the study is limited by the small sample size, the single-center design, and short follow-up duration.

## Conclusion

Pediatric stomas can be associated with unwanted complications, prolapse being one of the more common ones. Buttonpexy technique is a safe and viable first-line option to address the issue of prolapse, requiring minimal expertise, limiting anesthesia risks, and thus imparting great ease to both the patient and the surgeon.
